# Combination Effect of Epigenetic Regulation and Ionizing Radiation in Colorectal Cancer Cells

**DOI:** 10.1371/journal.pone.0105405

**Published:** 2014-08-19

**Authors:** Joong-Gook Kim, Jin-Han Bae, Jin-Ah Kim, Kyu Heo, Kwangmo Yang, Joo Mi Yi

**Affiliations:** 1 Research Center, Dongnam Institute of Radiological & Medical Sciences (DIRAMS), Busan, South Korea; 2 Department of Radiation Oncology, Korea Institute of Radiological and Medical Sciences, Seoul, Korea; Sapporo Medical University, Japan

## Abstract

Exposure of cells to ionizing radiation (IR) induces, not only, activation of multiple signaling pathways that play critical roles in cell fate determination, but also alteration of molecular pathways involved in cell death or survival. Recently, DNA methylation has been established as a critical epigenetic process involved in the regulation of gene expression in cancer cells, suggesting that DNA methylation inhibition may be an effective cancer treatment strategy. Because alterations of gene expression by DNA methylation have been considered to influence radioresponsiveness, we investigated the effect of a DNA methyltransferase inhibitor, 5-aza-2′-deoxycytidine (5-aza-dC), on radiosensitivity. In addition, we investigated the underlying cellular mechanisms of combination treatments of ionizing irradiation (IR) and 5-aza-dC in human colon cancer cells. Colon cancer cell lines were initially tested for radiation sensitivity by IR *in vitro* and were treated with two different doses of 5-aza-dC. Survival of these cell lines was measured using MTT (3-(4,5-dimethylthiazol-2-yl)-2,5-diphenyltetrazolium bromide) and clonogenic assays. The effects of 5-aza-dC along with irradiation on cell growth, cell cycle distribution, apoptosis, and apoptosis-related gene expression were examined. Combination irradiation treatment with 5-aza-dC significantly decreased growth activity compared with irradiation treatment alone or with 5-aza-dC treatment alone. The percentage of HCT116 cells in the sub-G1 phase and their apoptotic rate was increased when cells were treated with irradiation in combination with 5-aza-dC compared with either treatment alone. These observations were strongly supported by increased caspase activity, increased comet tails using comet assays, and increased protein levels of apoptosis-associated molecules (caspase 3/9, cleaved PARP). Our data demonstrated that 5-aza-dC enhanced radiosensitivity in colon cancer cells, and the combination effects of 5-aza-dC with radiation showed greater cellular effects than that of single treatment, suggesting that the combination of 5-aza-dC and radiation has the potential to become a clinical strategy for the treatment of cancer.

## Introduction

Epigenetics is the study of inheritable changes in gene expression or cellular phenotype caused by mechanisms other than changes in the underlying DNA sequences [1]. The epigenetic regulation of gene expression is mediated by mechanisms such as DNA methylation, modifications of histones, and positioning of the nucleosome along the DNA. Typically, DNA hypermethylation plays a critical role in the inactivation of genes involved in cell cycle regulation, DNA repair, apoptosis, cell signaling, transcription, and other cellular processes [2].

Aberrations in DNA methylation are frequently observed in many different cancer types [3], [4]. In particular, silencing of tumor suppressor genes or other cancer-related genes by aberrant DNA hypermethylation in promoter or regulatory regions contributes to tumorigenesis [5], [6]. Unlike genetic alterations, epigenetic events, including DNA methylation, are reversible, making epigenetic regulation extremely interesting from the point of view of developing new approaches to therapy. DNA hypermethylation can be reversed by DNA-demethylating agents. In addition, DNA methyltransferase (DNMT) inhibitors can restore the expression of genes silenced by DNA methylation. In recent years, the DNMT inhibitor, 5-aza-2′-deoxycytidine (5-aza-dC), has been shown to have anticancer activities in patients with leukemia, myelodysplastic syndrome, and several solid tumors [7], [8]. Although a single epigenetic therapy has not shown significant reactions against most solid tumors [9], preclinical studies suggest that a combination of epigenetic modifiers, such as DNMT inhibitors or histone deacetylase inhibitors, may be effective. In addition, combination of these epigenetic modifiers with conventional chemotherapeutics may also be effective. Therefore, these types of combinatorial therapies are being examined in clinical trials [10], [11]. However, few reports have investigated radiosensitivity associated with exposure to 5-aza-dC [12]–[15]. Recently, there has been growing interest in strategies using substances that regulate cellular radiosensitivity to increase tumor radiosensitivity. Therefore, in this study, we report the therapeutic potential of combining 5-aza-dC with ionizing radiation (IR) to increase radiosensitivity in colorectal carcinoma cells and examine the cellular mechanisms underlying these effects.

## Materials and Methods

### Cell culture and 5-aza-dC treatment

The human colorectal carcinoma cell lines: HCT116, SW480, Colo320, and RKO, which were obtained from the American Type Culture Collection (ATCC, VA, USA), as well as a double-knockout cells (DKO) for DNA methyltransferase-1 and DNA methyltransferase-3b in HCT116 cell line [16], which retains <5% genomic DNA methylation, were cultured at 37°C with 20% O_2_ and 5% CO_2_. The HCT116, DKO, and SW480 cells were maintained in McCoy's 5A medium (WelGENE, Daegu, Korea). Colo320 cells were maintained in RPMI medium (WelGENE). RKO cells were maintained in Dulbecco's Modified Eagle Medium (DMEM)(WelGENE) containing 10% fetal bovine serum (Hyclone, Logan, UT, USA) and 1% antibiotic-antimycotic (Gibco, Grand Island, NY, USA). The cells were treated with 5-aza-dC (0.5 or 1 µM; Sigma-Aldrich, St. Louis, MO, USA) once daily for 3 days (Figure S1).

### Ionizing irradiation (IR) exposure

Cells treated with 5-aza-dC for 3 days or non-treated control cells were exposed to gamma rays from a ^137^Cs-ray source (Eckert & Ziegler, Berlin, Germany) at a dose rate of 2.6 Gy/min. Following irradiation at doses of 2, 5, and 10 Gy, the cells were incubated for 3 days at 37°C, 20% O_2_, and 5% CO_2_.

### Clonogenic assay

HCT116, SW480, RKO, Colo320, and DKO cells were seeded in 6-well plates (5000 cells/well) and treated with 5-aza-dC and IR. Cells were cultured for 2 weeks. The culture medium was replaced with fresh medium every 2 days. The colonies were fixed and stained with 1.25% crystal violet, washed extensively to remove excess dye, and imaged using a MultiXpress C9250ND scanner (SAMSUMG, Seoul, Korea). Colonies with >50 cells/colony were counted using the Image-Pro Plus 7.0 software (Media Cybernetics, Rockville, MD, USA).

### Cell proliferation and cell viability assays

Cell proliferation was determined using the 3-(4,5-dimethylthiazol-2-yl)-2,5-diphenyltetrazolium bromide (MTT) assay. Cells (2×10^5^ cells/well) were seeded in 6-well plates and incubated at 37°C. After 48 h, cells were washed twice with phosphate-buffered saline (PBS), and 5 mg/mL MTT in PBS was added to each well for 4 h. After removing the MTT solution, a solubilization solution (dimethyl sulfoxide/ethanol, 1∶1) was added to each well to dissolve the formazan crystals. The absorbance at 570 nm was measured using a Paradigm microplate reader (Beckman Coulter, Fullerton, CA, USA). HCT116 cells treated with 5-aza-dC and/or irradiation were seeded at a concentration of 5×10^4^ cells/well in 6-well plates. After 24, 48, 72 and 96 h, the cells were harvested, diluted with a Trypan blue working solution, and counted to generate a growth curve.

### Tumor growth analysis

For *in vivo* evaluation of tumor growth, we used a subcutaneous tumor-bearing SCID mouse xenograft model generated by infecting cells. Female C.B-17 SCID mice were purchased from the Central Lab. Animals (Seoul, Korea). Six week-old female mice were divided into experimental groups (n = 3 in each group). Mice were injected subcutaneously into the right sides of the dorsal area with 5×10^6^ cells diluted in 100 µL of PBS. The tumor volumes were measured once per week. Tumor volume was estimated using the following equation: (short axis)^2^× (long axis) ×0.5.

### Flow cytometric analysis

Cell cycle analysis was conducted using propidium iodide (PI). Cells were trypsinized, washed with PBS, and fixed in 75% ethanol at 4°C for 1 h. Prior to analysis, cells were washed again with PBS, suspended in a cold PI solution with 1 mg/mL RNase, and incubated in the dark for 30 min at room temperature. Flow cytometry analysis was performed using a FACScan instrument (BD FACSAria; BD Biosciences, San Jose, CA, USA). Apoptosis of treated cells was assessed using an Annexin V/FITC Apoptosis Detection Kit (BD Biosciences). Briefly, all of the cells were seeded and treated in 100-mm dishes. Subsequently, the cells were washed twice with cold PBS. The cells were stained with phycoerythrin (PE) annexin V and 7-amino-actinomycin and incubated for 15 min in the dark. After staining, binding buffer was added to the cells, which were analyzed using the FACScan instrument. FACSDiva software (BD Biosciences) was used for data analysis.

### Caspase 3/7 activity assay

For the caspase 3/7 activity assay, the cells (5×10^3^ cells) were seeded with 100 µL of McCoy's 5A medium into a 96-well plate. After 24 h of incubation, 100 µL of the Caspase-Glu 3/7 assay kit (Promega, Madison, WI, USA) substrate and buffer mixture were added to each well. The cells and solutions were gently mixed for 30 min and incubated at room temperature for 1 h in the dark. Fluorescence activity was measured using a luminometer (ATTO, Tokyo, Japan). All of the assays were repeated three times and contained negative controls.

### DNA damage assay

DNA damage was determined using the OxiSelect Comet Assay Kit (Cell Biolabs, San Diego, CA, USA). 5-aza-dC- and/or IR-treated HCT116 cells (1×10^5^) were mixed with low-melting-point agarose (1∶10 ratio), and then the comet slide was filled with 75 µL of the mixture. The slides were incubated at 4°C for 30 min and then immersed in lysis buffer at 4°C in the dark for 1 h. The lysis buffer was then aspirated from slides, and slides were immersed in alkaline solution at 4°C in the dark for 30 min. Next, to eliminate the alkaline solution, slides were stored in Tris-acetate-EDTA (TAE) buffer for 5 min. The slides were placed in a horizontal electrophoresis chamber and subjected to electrophoresis with TAE buffer at 25 V for 20 min. The slides were dried and stained with DNA dye (CELL Biolabs). Comet tails were detected under a fluorescent microscope (Nikon, Tokyo, Japan).

### Western blot analysis

Cells were lysed in lysis buffer, and total cell lysates containing equal amounts of proteins were loaded onto 4–12% gels for sodium dodecyl sulfate-polyacrylamide gel electrophoresis and then transferred to polyvinylidene difluoride membranes (GE Healthcare Life Sciences, Piscataway, NJ USA). The membranes were blocked with 5% milk dissolved in PBS containing 0.02% Tween-20 and incubated overnight at 4°C with specific primary antibodies. The membranes were subsequently incubated with specific horseradish peroxidase conjugated secondary antibodies. Protein bands were visualized using a Fusion FX5 system (Vilber Lourmat, Eberhardzell, Germany). The following primary antibodies were used: anti-cleaved caspase 3 (Cell Signaling Technology, Danvers, MA, USA), anti-cleaved caspase 9 (Cell Signaling Technology), anti-cleaved PARP1 (Cell Signaling Technology), anti-survivin (Abcam, Cambridge, MA, USA), anti-p53 (Santa Cruz Biotechnology, CA, USA), and anti-α-actin (Sigma-Aldrich).

### Statistical analysis

The results were presented as means ± standard deviation. Statistical analyses were performed using Student's *t*-test. A *P*-value less than 0.05 was considered statistical significant.

### Ethic Statement

All animal protocols used in the current study were reviewed and approved by the Institutional Animal Care and Use Committee at Dongnam Institute of Radiological & Medical Sciences (DIRAMS-IACUC-11-005).

## Results

### Effects of 5-aza-dC on the radiosensitivity of colorectal cancer cell lines

**Figure 1:**
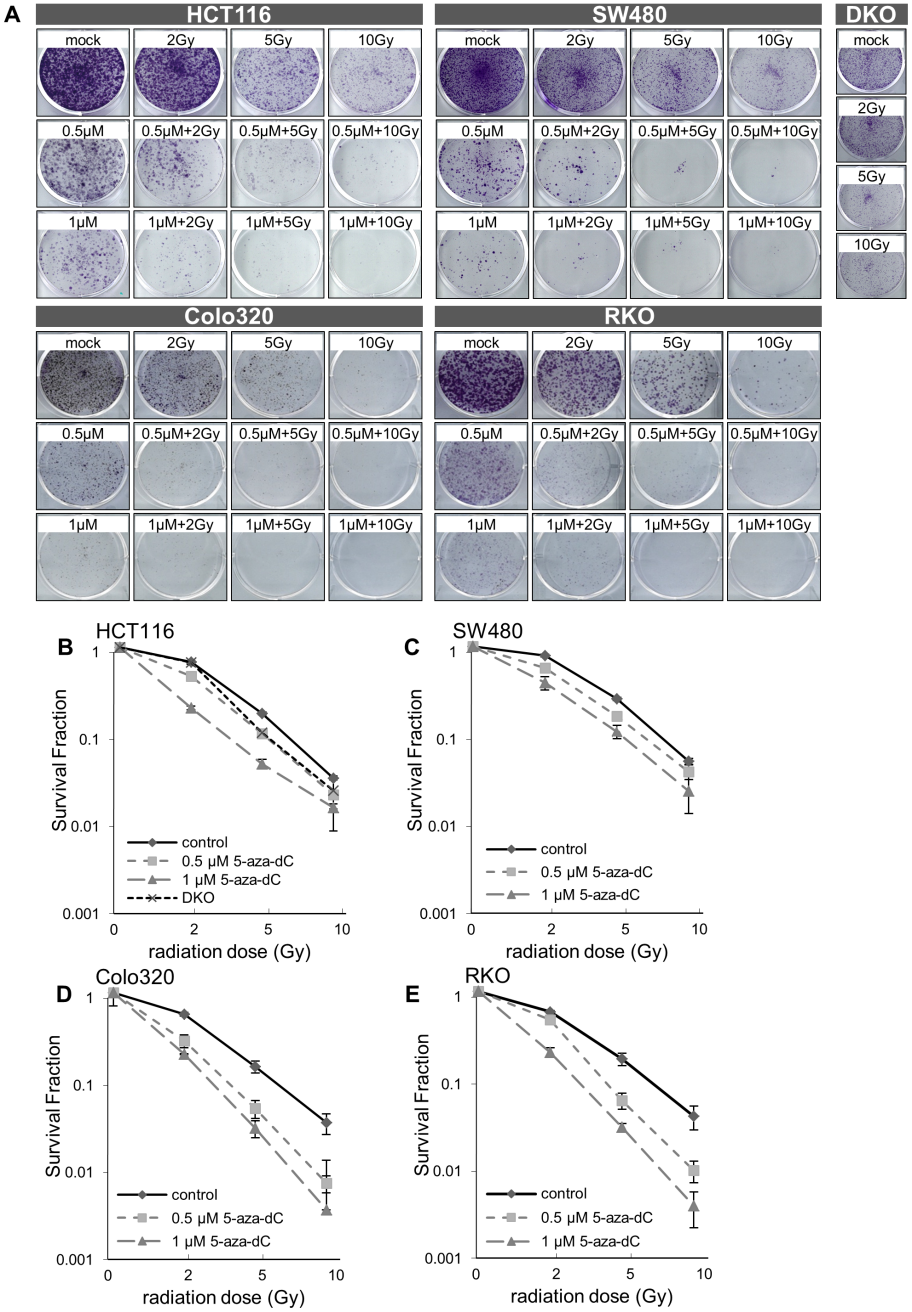
Effect of 5-aza-dC and ionizing radiation on colony formation in colon cancer cell lines. (A) Clonogenic assay of HCT116, SW480, Colo320, and RKO cells treated with 5-aza-dC (0.5 and 1 µM) and/or IR (2 Gy, 5 Gy, and 10 Gy). Clonogenic assay of irradiated (2 Gy, 5 Gy, and 10 Gy) DKO cells. Cells were seeded into 6-well plates (5000 cells/well) and treated with 5-aza-dC/IR. After 2 weeks, cultures were fixed with ethanol and stained with 1.25% crystal violet. Photographs of single colonies are also shown. (B–E) Survival fraction (SF) of HCT116/DKO (B), SW480 (C), Colo320 (D) and RKO (E) cells treated with 5-aza-dC (0.5 and 1 µM) and/or ionizing radiation (IR; 2 Gy, 5 Gy, and 10 Gy). The SF was calculated as mean colonies/seeded cells. The dose enhancement ratio was calculated as the ratio of the SF curve obtained by treatment with a combination of 5-aza-dC and IR to that obtained by treatment with IR alone. Data are expressed as the mean ± standard deviation of three independent experiments. *P*-values were calculated using Student's *t*-test. *P<0.05; **P<0.01; ***P<0.001.

To determine whether 5-aza-dC enhances the cellular sensitivity to IR, the colon cancer cell lines HCT116, SW480, RKO, Colo320, and DKO were exposed to 5-aza-dC at two different doses (0.5 µM and 1 µM) for 72 h before being exposed to three different IR doses (2 Gy, 5 Gy, and 10 Gy) (Figure S1). Next, a clonogenic assay was performed showing that 5-aza-dC could radiosensitize all of the colon cancer cell lines tested, and the combination of 5-aza-dC and IR was superior to treatment than 5-aza-dC alone. DKO cells, genetically inhibit DNMT1 and 3b, showed also growth suppression by IR with dose dependent manner (Figure 1A).

Radiation survival curves were generated for each cell line treated with 5-aza-dC and IR to understand whether irradiation can be sensitized by 5-aza-dC in colon cancer cell lines (Figure 1B–E). Using the dose required to generate a survival fraction (SF) of 0.5 as a reference, the dose enhancement rates (DERs) were estimated. Cells treated with 5-Aza-dC for 72 h before irradiation showed an increase in radiosensitivity, with a DER of: 1.19 (0.5 µM 5-aza-dC) and 1.41 (1 µM 5-aza-dC) in HCT116 cells, 1.23 (0.5 µM 5-aza-dC) and 1.42 (1 µM 5-aza-dC) in SW480 cells, 1.23 (0.5 µM 5-aza-dC) and 1.28 (1 µM 5-aza-dC) in Colo320 cells, and 1.14 (0.5 µM 5-aza-dC) and 1.31 (1 µM 5-aza-dC) in RKO cells (Figure 1B-E). Our data suggested that a lower dose of 5-aza-dC could radiosensitize colorectal cancer cells. Because 1 µM 5-aza-dC or 10 Gy of IR are cytotoxic, relatively low doses of 5-aza-dC (0.5 µM) and IR (2 Gy and 5 Gy) were chosen for further studies to examine the effects of combining a DNMT inhibitor, 5-aza-dC and IR.

### The combination of 5-aza-dC and IR induced growth suppression in colon cancer cells

**Figure 2:**
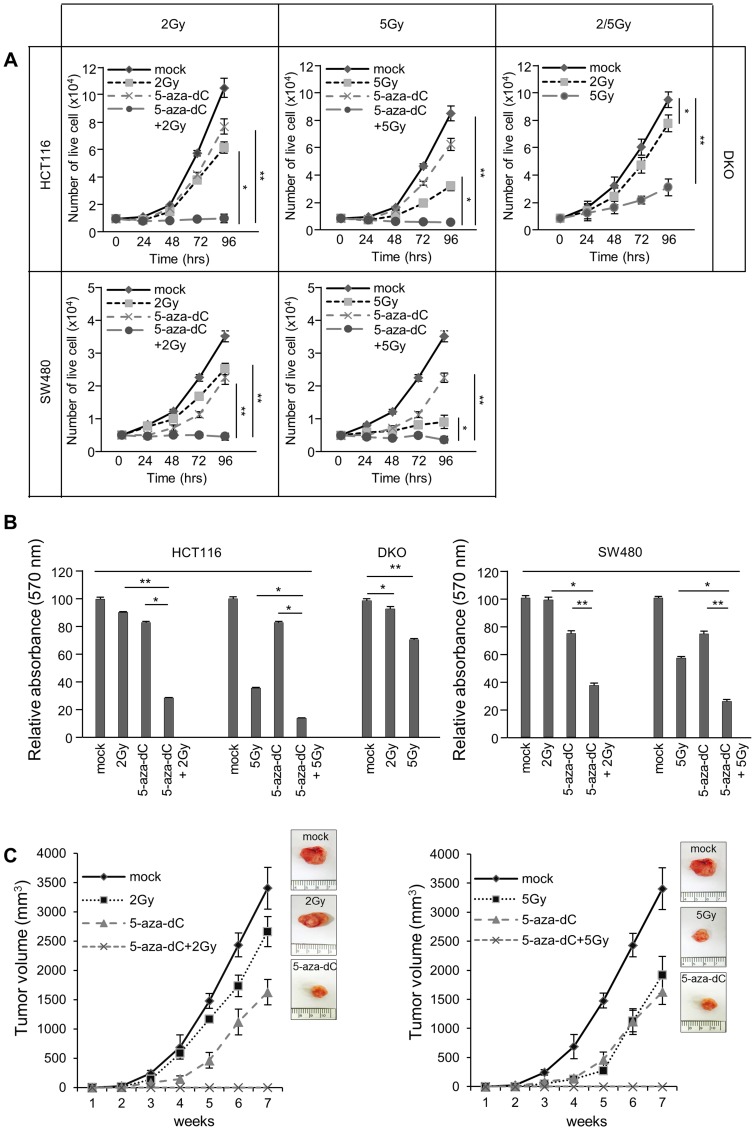
Growth inhibition in colon cancer cell lines in response to treatment with 5-aza-dC and irradiation. (A) Cell growth curves obtained using 0.5 µM 5-aza-dC and two different radiation doses (2 Gy and 5 Gy) in colon cancer cells (HCT116, DKO and SW480) and (B) MTT assays in colon cancer cells treated with 5-aza-dC (0.5 µM) and/or irradiation (2 Gy and 5 Gy). Data are expressed as the mean ± standard deviation of triplicate experiments. (C) Tumor growth following 5-aza-dC (0.5 µM) treatment and/or irradiation (2 Gy and 5 Gy) in SCID mice. HCT116 cells (5×10^6^ cells) that had been treated with 5-aza-dC (0.5 µM) and irradiated (2 Gy and 5 Gy) were injected subcutaneously into SCID mice (n = 4), and the average tumor size was measured once weekly for 7 weeks. *P*-values were calculated using Student's *t*-test. *P<0.05; **P<0.01.

We analyzed cell proliferation in HCT116 cells treated with 5-aza-dC or IR alone or with the combination of 5-aza-dC and IR. The growth curves showed that treatment with a combination of 5-aza-dC and IR (2 Gy and 5 Gy) resulted in statistically significant growth inhibition in HCT116 and SW480 cells at each time point examined (24, 48, 72, and 96 h) compared with that in control cells, in cells treated only with 5-aza-dC, or in cells treated only with IR (2 Gy and 5 Gy) (Figure 2A; *P*<0.05). In addition, we carried out the MTT assay in both HCT116 and SW480 cells that had been treated with or without 5-aza-dC (0.5 µM) and then irradiated them with 2, 5, and 10 Gy, respectively. The absorbance from the assay performed on cells treated with a combination of 5-aza-dC and IR was significantly lower (*P*<0.05) than that from cells treated with 5-aza-dC or IR alone (Figure 2B). We also performed growth curve analysis and the MTT assay in DKO cells, which also showed a decrease of growth suppression as well as proliferation, depending on the radiation dose (Figure 2A and B). Thus, these results indicate that the effects of 5-aza-dC and IR on growth inhibition are additive. Based on our *in vitro* data of significant growth inhibition in cells treated with the combination of 5-aza-dC and IR, we were interested in determining whether these effects could be observed *in vivo*. To this end, HCT116 cells were exposed to 5-aza-dC (0.5 µM) for 72 h before exposure to IR (2 Gy and 5 Gy), and then were subcutaneously injected into SCID mice. Figure 2C showed significantly delayed tumor growth with the combination treatment of 5-aza-dC and IR (2 Gy and 5 Gy) compared with single treatment with either 5-aza-dC or IR. In addition, the volume of the xenografts from cells treated with both 5-aza-dC and IR were reduced compared with those from cells treated with either 5-aza-dC or IR alone.

**Figure 3:**
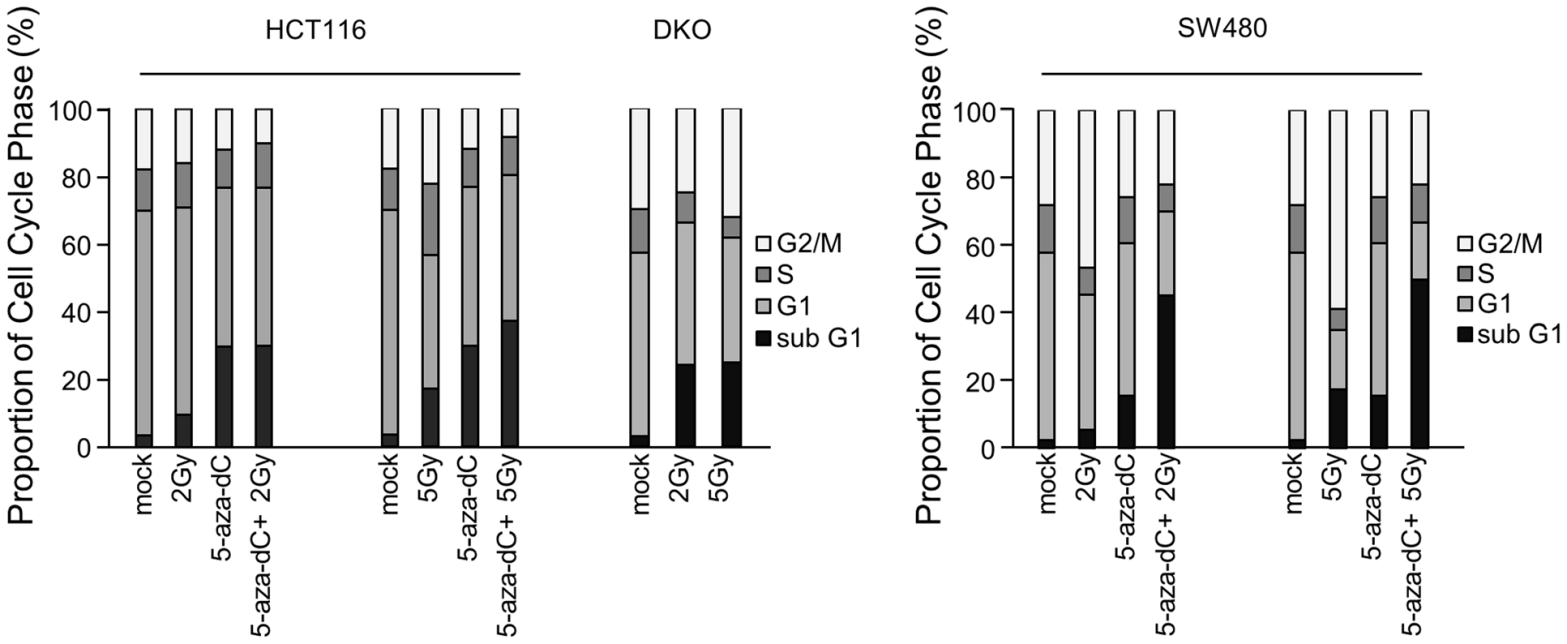
Cell cycle distributions of colon cancer cell lines in response to 5-aza-dC and irradiation exposure. Colon cancer cells (HCT116, DKO and SW480) treated with 5-aza-dC (0.5 µM) and/or irradiation (2 Gy and 5 Gy) were stained with propidium iodide and analyzed using a FACS flow cytometer. Columns show the proportion of cells in each cell cycle phase. Black column, sub-G1 phase; bright gray column, G1 phase; dark gray column, S phase; white column, G2-M phase.

To elucidate the mechanisms of growth inhibition by 5-aza-dC, IR, and the combination treatment, we used flow cytometry analysis to determine whether growth inhibition was associated with cell cycle changes. Cell cycle distribution analysis showed that the proportion of treated cells in the G1, S, and G2-M phases was not different from the control cells except that there was an increase in the proportion of cells in the sub-G1 phase, suggesting an increase in apoptosis (Figure 3). The proportion of HCT116 cells treated with 5-aza-dC or IR (2 Gy) alone in the sub-G1 phase of cells was eight fold (5-aza-dC), two fold (IR, 2 Gy), and four fold (5 Gy) greater than that of control cells and over eight fold greater in cells treated with 5-aza-dC and IR than in control cells. Interestingly, unlike HCT116 cells, SW480 cells showed G2/M arrest after IR alone (2 Gy and 5 Gy) compared with controls [2-Gy G2/M fraction: 46.77% (vs. 28.03% in control cells); 5 Gy G2/M fraction: 58.88% (vs. 22.03% in control cells)]. However, the sub-G1 phase of cells treated with 5-aza-dC or IR (2 Gy) alone was six fold (5-aza-dC), two fold (2 Gy), and seven fold (5 Gy) greater than control cells and an over 19-fold increase in cells treated with 5-aza-dC and IR than in control cells. We also confirmed that the sub-G1 phase of cells irradiated with 2 Gy or 5 Gy compared with control cells was increased in DKO cells. Therefore, we conclude that the growth inhibitory effects of 5-aza-dC or IR are due to an increase in apoptosis.

### The combination of 5-aza-dC and IR contributes to the induction of apoptosis in colon cancer cells

**Figure 4:**
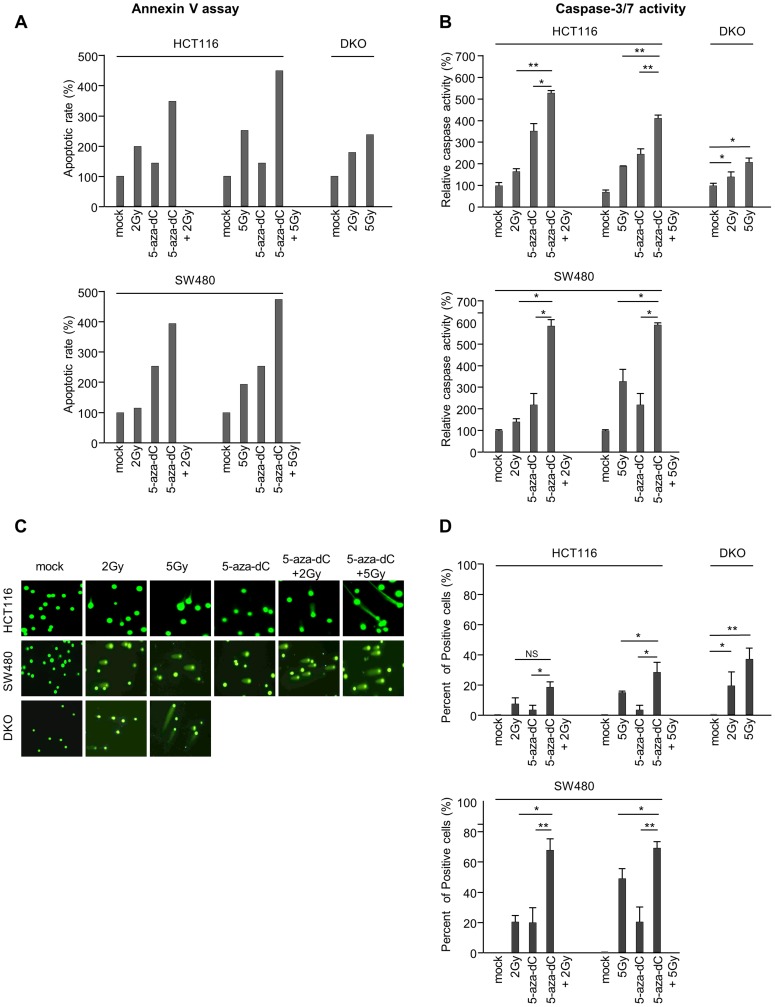
Induction of apoptosis in colon cancer cells by 5-aza-dC or IR, both alone and in combination. (A) The levels of apoptosis were measured using annexin V and 7-amino-actinomycin and analyzed using a FACS flow cytometer. The levels of apoptosis in HCT116, DKO and SW480 cells treated with 5-aza-dC (0.5 µM) and/or irradiation (2 Gy and 5 Gy) were expressed as percentages of the total cell population at both the early and late stages of apoptosis. (B) The activities of caspases 3 and 7 were determined using the Caspase-Glo assay and were represented as percentages of HCT116, DKO and SW480 cells treated with 5-aza-dC (0.5 µM) and/or irradiation (2 Gy and 5 Gy) compared with untreated cells. The graph represents data (mean ± standard deviation) from three independent experiments. (C) Representative micrographs of fluorescent DNA stain using the comet assay. DNA fragmentation by the comet assay in HCT116, SW480 and DKO cells treated with 0.5 µM 5-aza-dC and/or irradiation (2 Gy and 5 Gy). (D) Quantification of DNA damaged cells represents the mean of three random microscopic fields per sample, and the error bars represent ± standard deviations. NS indicates not significant. *P*-values were calculated using Student's *t*-test. *P<0.05; **P<0.01.

To clarify the induction of apoptosis by 5-aza-dC combined with IR in HCT116 and SW480 cells, cells were double stained with fluorescein isothiocyanate-labeled annexin V and PI. The level of apoptosis induced by 5-aza-dC combined with IR was greater than that by IR (1.7-fold for 2 Gy or 1.8-fold for 5 Gy) or 5-aza-dC alone (>2.4 fold) in HCT116 cells. Interestingly, we observed similar results in elevated apoptosis levels induced by the combination treatment with 5-aza-dC and IR compared with IR (3.4-fold for 2 Gy or 2.4-fold for 5 Gy) or 5-aza-dC (>1.5-fold) alone in SW480 cells (Figure 4A). Furthermore, we investigated the cellular mechanisms underlying the apoptotic effects of the combination of 5-aza-dC and IR. One of the most common signaling cascades involved in apoptosis is the activation of the highly apoptosis specific family of caspases, which, once activated, initiate cell death by cleaving and activating effector caspases driving apoptosis [17]. To determine whether caspases mediated the effects of 5-aza-dC and IR, we measured the activities of caspases 3 and 7, which are key effectors for apoptosis in mammalian cells [18]. In both cell extracts treated with 5-aza-dC and IR, either alone or in combination, the activities of caspases 3 and 7 were greatly increased in cells treated with 5-aza-dC and IR (2 Gy and 5 Gy) compared with those in cells treated with IR or 5-aza-dC alone (Figure 4B). These results correspond with increased in apoptosis caused by the combination treatment of 5-aza-dC and IR and is shown in Figure 4A. In both analyses, DKO cells were also shown increasing the level of apoptosis by IR. These results are strongly supported by longer comet tails observed in the comet assay, indicating greater amount of cellular DNA damage in cells treated with a combination of 5-aza-dC and IR compared with control cells or cells treated with 5-aza-dC or IR alone (Figure 4C and D).

**Figure 5:**
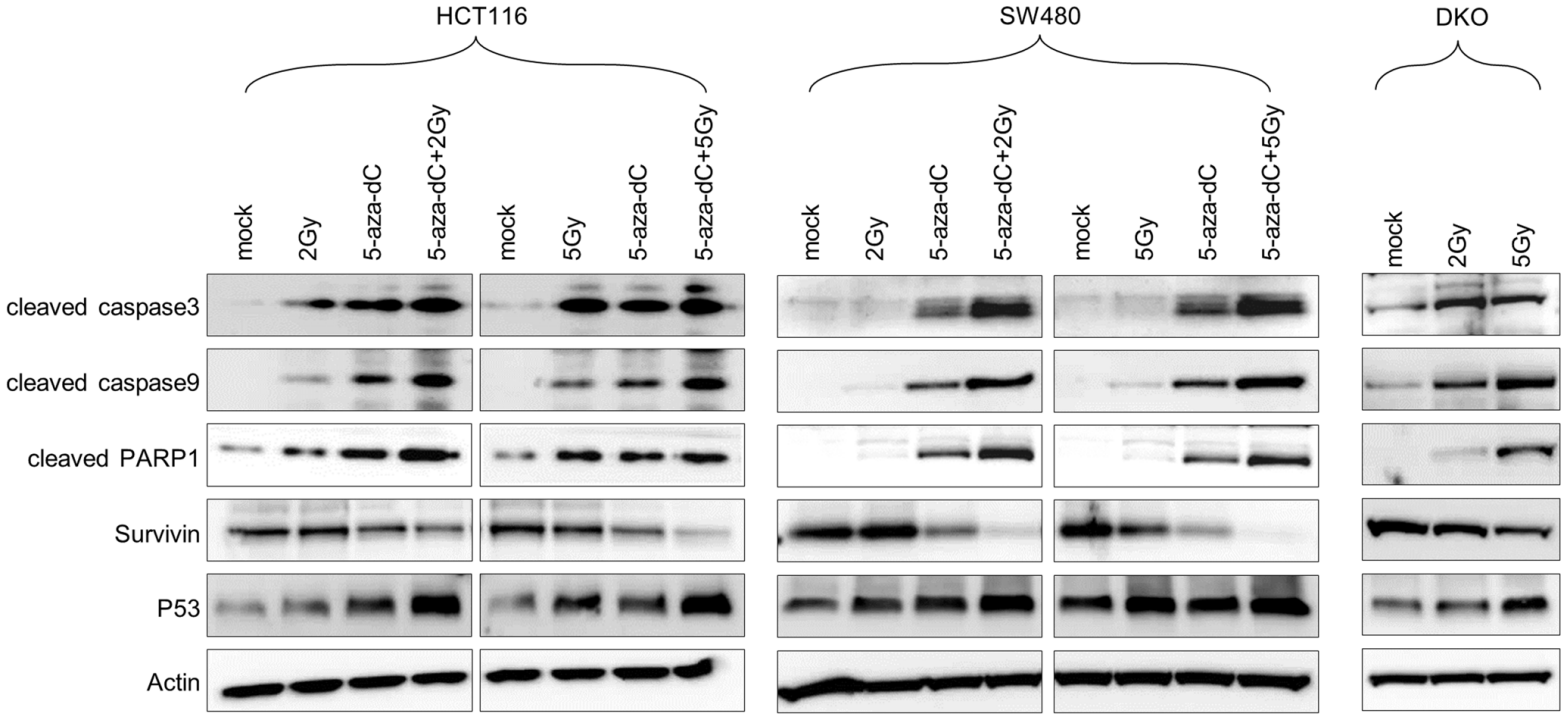
Expression levels of apoptosis-associated proteins in colon cancer cells following treatment with 5-aza-dC or IR, both alone and in combination. Western blot analysis for the expression of apoptosis-associated proteins (cleaved caspase 3, cleaved caspase 9, cleaved PARP1, survivin, and p53) in HCT116 and SW480 cells treated with 5-aza-dC (0.5 µM) and/or irradiation (2 Gy and 5 Gy), as well as in irradiated DKO cells, using cleaved caspase 3, cleaved caspase 9, cleaved PARP1, survivin, and p53 antibodies.

Caspases 3 and 9 are also known to be involved in radiation-induced apoptosis [19]. Thus, we used western blotting to confirm the activation levels of caspases 3 and 9 in cells treated with 5-aza-dC and/or IR. Figure 5 shows higher levels of activated caspases 3 and 9 in HCT116, DKO, and SW480 cells treated with 5-aza-dC or IR alone compared with those in control cells. Interestingly, western blotting, in the three, tested colon cancer cell lines, revealed that the protein levels of caspases 3 and 9 were also increased in cells treated with a combination of 5-aza-dC and IR compared with cells treated with either agent alone or in controls cells. The level of cleaved PARP1, which is another key effector of apoptosis induction [20], [21], was also increased in cells treated with 5-aza-dC or IR alone compared with control cells, and even more increased in cells treated with a combination of 5-aza-dC and IR. By contrast, the protein expression levels of survivin were decreased in cells treated with 5-aza-dC and IR alone and in combination compared with those in control cells. In addition, we tested the level of p53 as well. Interestingly, the p53 expression level increased in cells treated with a combination of 5-aza-dC and IR than in cells treated with 5-aza-dC alone. This data is agreement with previous report which is cells expressing wild type of p53 are better sensitive IR than mutant type of p53 [14] . These protein expression patterns were confirmed in irradiated DKO cells. Taken together, our data suggest that the combination of 5-aza-dC and IR synergistically induces a higher level of apoptosis compared with single treatment using 5-aza-dC or IR in several colon cancer cells.

## Discussion

IR exposure results in the simultaneous activation or downregulation of multiple signaling pathways that play critical roles in cell type-specific control of survival or death. IR is a well-known genotoxic agent and human carcinogen that induces cellular damage through direct and indirect mechanisms [22]. Recently, many studies have focused on the molecules and processes that influence the response of cells to IR. Many different types of molecules are known to increase radiosensitivity by affecting cell cycle checkpoints, DNA repair, gene transcription, and apoptosis. The most recent studies have suggested that epigenetic mechanisms such as histone modification and DNA methylation are associated with gene silencing and may be involved in regulating radiosensitivity in cancer cells. A number of previous studies have reported that several histone deacetylase inhibitors are cytotoxic and can sensitize tumor cells to radiotherapy. However, scant information is available regarding the effects of DNMT inhibitors on radiosensitization [13], [23]. Moreover, 5-aza-dC has been demonstrated to have little activity in solid tumors as a single agent [24], [25], and little is known concerning the molecular or cellular mechanisms underlying the radiosensitivity induced by epigenetic inhibitors. Combining epigenetic drugs with radiotherapy is particularly interesting, in this context, and has demonstrated improved efficacy both *in vitro* and *in vivo* in several solid tumors [13]–[15], [26]. In the present study, we investigated the cellular effects of the DNMT inhibitor, 5-aza-dC, and IR, both alone and in combination, on colon cancer cells. We found that 5-aza-dC demonstrated additive effects on growth inhibition combined with IR, suggesting that 5-aza-dC might be a useful radiation sensitizer in colon cancer treatment. One thing that we need to consider further based on our results, DKO cells, genetic model system for inhibition of DNA methyltransferase, do not seem to have more strong growth suppressive effect with IR than using pharmacological model system that we treated 5-aza-dC with IR.

Since our results indicate that this effect is mediated by the induction of apoptosis, apoptosis has been previously regarded as a potential mechanism for radiosensitization. Several different results have been reported regarding the radiosensitizing effects of DNMT inhibitors. Dote et al. previously reported that the combination of IR and zebularine did not significantly increase apoptosis [13]. By contrast, Qiu et al. demonstrated that 5-aza-dC induced radiosensitization in certain gastric cancer cell lines and caused an increase in apoptosis, which was accompanied by enhanced expression of *p53*, *RASSF1*, and *DAPK* gene families [14]. In our study, 5-aza-dC increased the level of apoptosis in HCT116 and SW480 cells, a finding that was consistent with results from Qiu et al. Very interestingly, Qiu et al. also suggested that gastric cancer cell lines expressing wild-type p53 are more sensitive to combination therapy with IR and 5-aza-dC compared with those expressing mutant p53. The HCT116 cell line used in this study expresses wild-type p53, and we observed that the p53 level increased in response to 5-aza-dC treatment both with and without IR. In addition, the p53 expression level increased in cells treated with a combination of 5-aza-dC and IR than in cells treated with 5-aza-dC alone. However, unlike in HCT116 cells, no effect was shown on the p53 level in SW480 cells, which expresses mutant type p53 (Figure 5). p53 has been classically described as a mediator of IR cytotoxicity and acts by promoting either cell cycle arrest or apoptosis [27], [28]. Previous studies have reported that 5-aza-dC induces p53 expression, which is associated with inhibition of cell proliferation in wild-type p53 cells but not in mutant p53 cells in prostate cancer [29], [30]. However, because only a few cell lines and p53-associated molecules were examined in these studies, further research into the possible association of 5-aza-dC with p53 is necessary. Further investigation is also required to identify additional epigenetic alterations associated with radiosensitivity. There are limited studies on the role of DNA methylation in resistance to IR. One previous study indicated that treatment with 5-aza-dC causes global hypomethylation, which has a radiosensitizing effect [23]. Therefore, definitive studies should be conducted to determine whether IR has an effect on site- or gene-specific DNA methylation in cancer (manuscript in preparation).

In this study, cell cycle analysis was not significantly altered by single treatment with 5-aza-dC or IR, as well as by the combination treatment with both, except for the proportion of cells in the apoptotic sub-G1 phase. The proportion of cells in the G2/M phase detected in HCT116 and SW480 cells was not significantly changed by 5-aza-DC alone, a finding that is in agreement with other previous studies [31], [32]. Although there is accumulation of the G2/M phase by treatment with radiation alone in SW480, a discrete apoptotic sub-G1 peak appeared in the combined treatment. Thus, it is likely that, after combination treatment with 5-aza-dC and IR, 5-aza-dC may modify the apoptotic portion of cells to some extent.

Other groups have previously reported that 5-aza-dC enhanced radiosensitivity in various cancer types [14], [23], [26]. In the present study, a low dose of 5-aza-dC (0.5 µM) was used in combination with IR. As shown in Figure 1, although we tested treatment using 1 µM 5-aza-dC in combination with different doses of IR, we observed that a lower dose of 5-aza-dC (0.5 µM) and a nontoxic level of IR (2 Gy) are sufficient to induce anticancer effects in colorectal cancer cell lines. Although Schuebel et al. reported that 5 µM 5-aza-dC was required to reactivate the expression of most silenced genes [33], we found that 0.5 µM 5-aza-dC can reactivate most of the key genes regulated by promoter DNA methylation in HCT116 cells (data not shown).

In conclusion, a lower dose of 5-aza-dC can act as a radiation sensitizer to induce apoptosis in combination with IR. We have confirmed that treatment of HCT116 and SW480 cells with a combination of 5-aza-dC and IR increased the level of apoptosis and contributed to an anticancer effect. Our results strongly suggest that combining 5-aza-dC and IR may be a potential cancer treatment strategy.

## Supporting Information

Figure S1 **Experimental procedures for this study.** Human colorectal carcinoma cell lines (HCT116, SW480 and DKO) were seeded and treated with 5-aza-dC (0.5 or 1 µM) for 72 h prior to irradiation with gamma rays (2, 5, or 10 Gy). Twenty-four hours after irradiation, the cells were assayed for colony formation, cell proliferation, cell viability, tumor growth, cell cycle, annexin V, and the activity levels of caspases 3 and 7. In addition, comet assays and western blots were also performed.
(DOCX)
