# Supplementary figures and images for: Combination Effect of Epigenetic Regulation and Ionizing Radiation in Colorectal Cancer Cells
Source: PLoS One. 2014 Aug 19;9(8):e105405. doi: 10.1371/journal.pone.0105405 (PMC4138159; doi:10.1371/journal.pone.0105405)

**Supporting Information**

**Figure S1.**


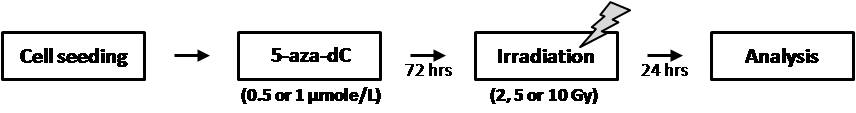

Supplement: Figure S1 — Experimental procedures for this study. Human colorectal carcinoma cell lines (HCT116, SW480 and DKO) were seeded and treated with 5-aza-dC (0.5 or 1 µM) for 72 h prior to irradiation with gamma rays (2, 5, or 10 Gy). Twenty-four hours after irradiation, the cells were assayed for colony formation, cell proliferation, cell viability, tumor growth, cell cycle, annexin V, and the activity levels of caspases 3 and 7. In addition, comet assays and western blots were also performed. (DOCX) [file pone.0105405.s001.docx]
